# Surgical consent during the COVID-19 pandemic

**DOI:** 10.1016/j.amsu.2020.10.011

**Published:** 2020-10-09

**Authors:** O. Rotimi, K. Beatson, A. Aderombi, W. Lam, O. Bajomo, N. Kukreja

**Affiliations:** General Surgery Department, Windmill Road, Medway Maritime Hospital, Gillingham, Kent, ME7 5NY, United Kingdom

**Keywords:** Coronavirus, Surgical guidance, Audit, Consent

## Abstract

**Background and aims:**

During the COVID-19 pandemic, surgical practice may deviate with operative and non-operative management considered. Appropriate discussion of options with patients is paramount to quality surgical care. Intercollegiate and EAES guidelines recommend discussing and documenting risk of COVID-19 exposure in the consent process for patients undergoing surgery.

**Materials and methods:**

Closed-loop audit of consent forms for patients undergoing emergency and elective surgical procedures. Interventions implemented included education of wider surgical teams. Data was collected during a one-week period for each cycle and analysed using Chi-squared test.

**Results:**

In cycle 1, 6/17 (35.3%) case notes documented discussion of COVID-19 risk. Following intervention, compliance improved to 23/29 (79.3%) cases in cycle 2 and 33/45 (73.3%) cases in cycle 3.

**Conclusion:**

Pre-intervention, our consenting practice was non-compliant. Our interventions led to significant and sustained improvements in practice. We recommend provision of wider surgical team education to facilitate good consenting practice.

## Highlights

• Surgical consent practice has changed as a result of COVID-19.
• Patients should be consented for COVID-19 as this risk is now pertinent to all patients listed for surgery.
• Simple interventions can be extremely effective in improving long-term consenting practice.

## 1 Introduction

The World Health Organisation declared COVID-19 a pandemic on 11 March 2020. At the time of writing this paper, the number of cases worldwide has increased 200-fold, and the United Kingdom (UK) now has the highest number of confirmed deaths in Europe at 41,498 people [1]. As the health service endeavours to mitigate the transmission of the virus, surgical practice may deviate. Non-operative intervention is explored where appropriate, operations postponed where safe, and open procedures considered over laparoscopy to reduce occupational exposure [2]. With concern over asymptomatic and nosocomial transmission [3], post-operative inpatient admission may expose the patient to COVID-19. The European Association of Endoscopic Surgeons (EAES) and UK surgical colleges have published guidance on general surgery during the pandemic. These recommend discussion and documentation of the risk of COVID-19 in planning and consent [4,5]. The aim of this audit is to assess our consent practice at a district general hospital against these guidelines during the COVID-19 pandemic.

## 2 Methods

### 2.1 Design

Closed loop retrospective and prospective cross-sectional analysis of consent for patients listed for emergency and elective surgical cases.

Independent observers reviewed the consent forms for all elective and emergency patients over a 7-day period during cycle 1, 2 and 3. Evidence of discussion of risks to specific COVID-19 on the consent form was sought. There was a one-week interval following the intervention before data collection for cycle 2. A 3-month interval following completion of cycle 2 before data collection for cycle 3 was initiated.

### 2.2 Participants

All emergency and elective patients who have undergone operations in General Surgery department were included, comprising of cases in Vascular, Breast and General/Colorectal Surgery.

### 2.3 Interventions

Following results from cycle 1, two interventions were implemented. A morning briefing in our department was held during the pandemic. Dissemination of findings during one of these sessions allowed education of the wider surgical team through open discussion. Additionally, visual prompts of guidance were strategically placed within the department to reinforce application of guidance. Following cycle 2, the same interventions were delivered in our audit meeting following current social distancing guidance. This was conducted 1 month prior to data collection for cycle 3.

### 2.4 Analysis

Data analysis included descriptive statistics and Chi-squared test.

## 3 Results

### 3.1 Participants and baseline demographics

The data above shows the demographics of each cycle of this audit (see Table 1).

**Table 1:** Patient demographics.

	Cycle 1	Cycle 2	Cycle 3
**Male: Female ratio**	8 : 9	16 : 13	28 : 17
**Median ± SD age**	67 ± 17.6	53 ± 17.8	56 ± 19.2

### 3.2 Surgical cases

**Fig. 1:**
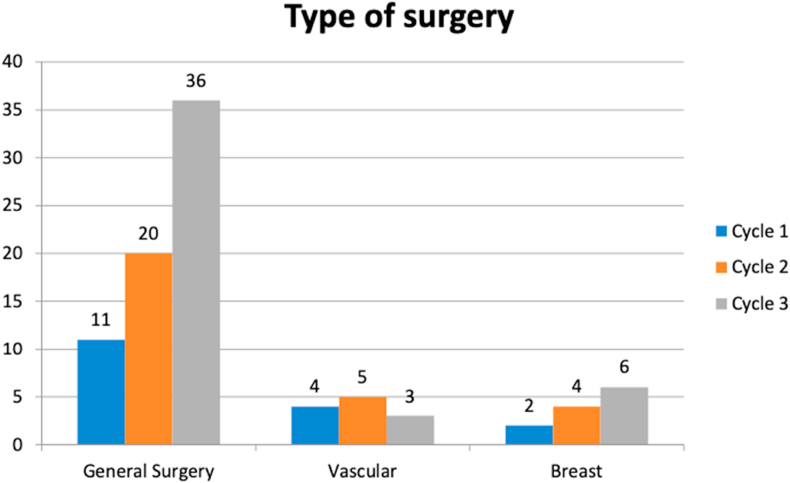
Breakdown of surgical cases by specialty.

The vast majority of cases in cycle 1, 2 and 3 were general surgical procedures (65%, 69% and 80% respectively), in keeping with the hospital's norm. Emergency cases were more prevalent in our study than elective cases during cycle 1 and 2, however elective cases became more prevalent in cycle 3. Emergency cases represented 59% of cases in cycle 1 and cycle 2. In cycle 3, emergency cases represented 38% of cases (see Table 2 and Fig. 1).

**Table 2:** Table showing Elective vs Emergency cases.

	Cycle 1	Cycle 2	Cycle 3
**Total number of cases**	17	29	45
**Total Elective cases**	7	12	28
**Total Emergency cases**	10	17	17

#### 3.2.1 Consenting practice

Of all emergency operations, 10% (n = 1/10) of pre-intervention cases had COVID-19 documented in their consent form. The documentation of COVID-19 in consent forms in emergency cases improved significantly to 94% (n = 16/17) post-intervention in cycle 2 and 88% (n = 15/17) post-intervention in cycle 3.

The proportion of COVID-19 documented in consent forms for elective cases in all subspecialties decreased from 71.4% (n = 5/7) pre-intervention to 66.7% (n = 8/12) post-intervention in cycle 2. However in cycle 3, the consenting practice of all subspecialties increased to 64.2% (n = 18/28). For elective and emergency procedures within each subspecialty, compliance improved in general/colorectal from 27% (n = 3/11) to 95% (n = 19/20) in cycle 2 and 72.2% (n = 26/36) in cycle 3. Consenting practice compliance improved in vascular surgery from 25% (n = 1/4) to 100% (n = 5/5) in cycle 2 and 66.7% (n = 2/3) in cycle 3. Breast surgery compliance reduced from 100% (n = 2/2) to 0% (n = 0/4) in cycle 2 and increased to 83.3% (n = 5/6) in cycle 3.

**Fig. 2:**
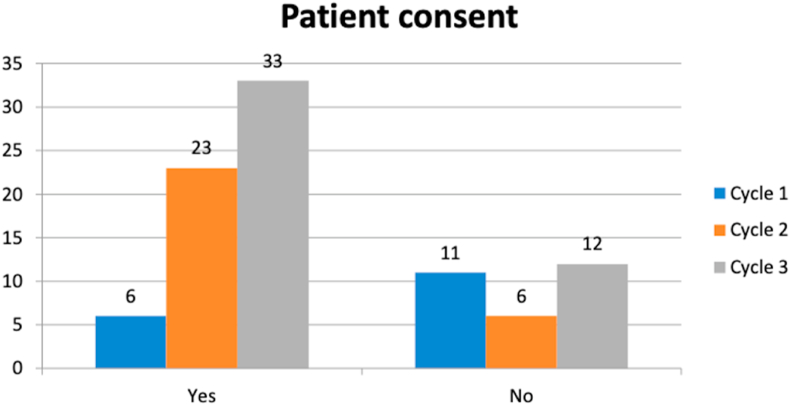
Patients with COVID-19 written in consent documentation.

**Fig. 3:**
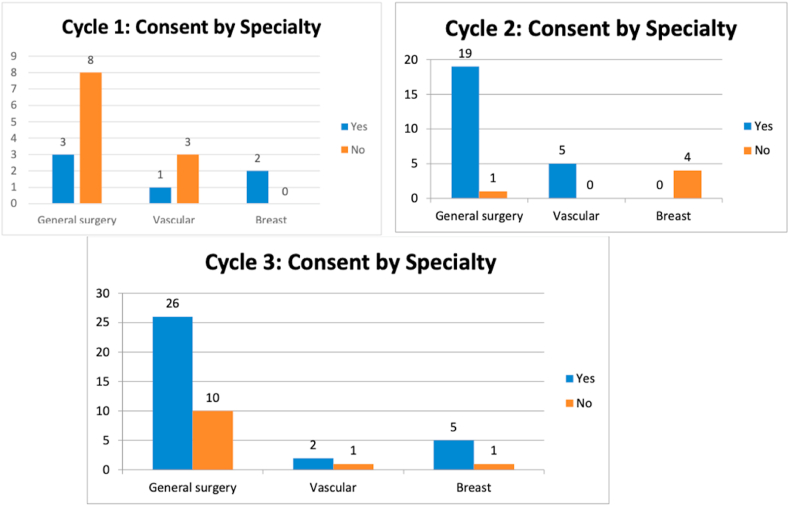
Patients with COVID-19 written in consent documentation by specialty.

**Fig. 4:**
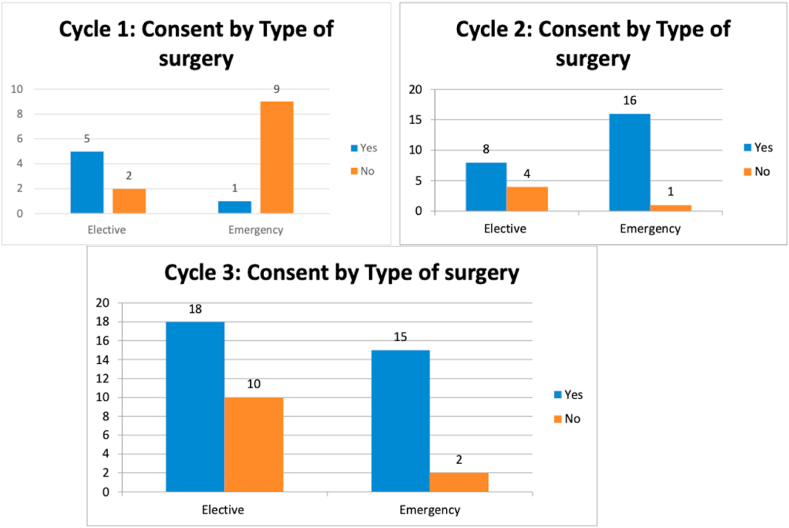
Patients with COVID-19 written in consent documentation by type of surgery.

Overall, pre-intervention consenting practice was approximately 35% (n = 6/17) of all cases documented in the department. Post intervention consenting practice improved significantly to approximately 79% (n = 23/29) in cycle 2 and 73.3% (n = 33/45) in cycle 3 (see Fig. 2, Fig. 3, Fig. 4).

## 4 Discussion

COVID-19 has posed challenges to the provision of surgical services, hence much of the guidance available has focused on re-organisation of elective surgery, prevention of disease transmission and appropriate triaging of surgical procedures based on COVID-19 risk and surgical pathology [6,7]. These measures aim to minimise exposure to staff and patients. Therefore, the risk and implications of viral transmission in hospital should be discussed when gaining consent prior to operative management in line with Montgomery principles.

We found our consenting practice non-compliant, particularly with emergency procedures. There is evidence in the literature that challenges such as time constraints, impaired patient consciousness due to trauma or pain and sudden change in circumstance that may contribute to poorer consent practice [2,8]. The stresses of busy on-call shifts can result in surgeons falling back on habit and discuss risks mechanistically. The oversight of not undertaking consent discussions on COVID-19 infection could have medicolegal implications, particularly in cases where non-operative management or postponement of surgery may be considered. In cases where operative management is pursued, the incidence of COVID-19 post-operatively is suggested to be as high as 17% when COVID-19 was more prevalent during the peak of the pandemic [9]. Therefore, it is a significant risk that all patients undergoing surgery should be consented for.

Our interventions are simple and have led to significant improvement in practice overall, and within General/Colorectal, Vascular and Breast surgery. They have been shown to have long term impacts as adherence to audit standards have been maintained. There is potential to maintain and build on gains through default documentation of COVID-19 and organisational related risks in consent forms [10]. Our interventions, in addition to clear frameworks on detailing these risks, could further improve compliance to surgical guidance as well as the quality of consent discussions.

As there is uncertainty whether future surges in COVID-19 cases may occur as government measures relax, continued consideration should be made to our discussions with patients as to risks of surgery. Given variation in consent practice between centres [11], our findings may represent an area for improvement in other surgical departments and specialties.

### 4.1 Limitations

There are limitations to our audit. First, there is a limited sample size in cycles 1 and 2. The sample sizes were a result of a significant reduction in elective procedures and patient presentation to emergency services within our trust during the pandemic period. Furthermore, this audit was designed to be a short cross-sectional analysis to determine adherence to guidance rather than ensure statistical power.

This study highlights that simple interventions can improve consent discussions surrounding COVID-19. However, it assumes documentation is evidence of good quality discussion of risk and implications. Documentation of a risk may not correlate with the quality of information provided [8]. These limitations cannot be eliminated without being present in or recording all consent discussions them which may lead to logistical and confidentiality issues. The authors acknowledge these limitations exist and should be considered when interpreting our audit results. Interestingly, there has been recent guidance provided by the Royal College of Surgeons of England (RCSEng). We recognise the RCSEng tool recommending considerations that should be discussed when consenting practice during the COVID pandemic as an important step towards improving the patient-surgeon relationship and discussions around surgery during these uncertain times [12]. Going forward, the authors suggest this could be adapted into a user-friendly leaflet format to inform patients while minimising anxiety around hospital admissions at this time.

## 5 Conclusion

The impact of COVID-19 on surgical services has been significant, however, guidance is in place to maintain quality of care. There is specific guidance on consenting patients on the risk associated with COVID-19, but factors during provision of emergency care may limit the frequency of this guidance being followed. Owing to uncertainty with how the current pandemic will unfold, we recommend simple interventions such as education of the surgical team, visual prompts and amended consent forms to improve adherence to surgical guidance in the era of COVID-19.

## Funding

This research did not receive any specific grant from funding agencies in the public, commercial, or not-for-profit sectors.

## Conflicts of interest

None to declare.

## Ethical approval

Approved by Audit Department.

## Author contributions

OR developed the concept for the project, drafted and made corrections to the manuscript. KB developed the concept of the project and made corrections to the manuscript. OB, AA, WL and NK were involved in data collection and revision of the manuscript.

## Provenance and peer review

Not commissioned, externally peer reviewed.

## Consent

Not required.

## Registration of Research Studies

Name of the registry: ClinicalTrials.gov

Unique Identifying number or registration ID: NCT04556604

Hyperlink to your specific registration (must be publicly accessible and will be checked): https://clinicaltrials.gov/ct2/results?cond=&term=NCT04556604&cntry=&state=&city=&dist=

## Guarantor

Oloruntobi Rotimi.
